# AdpA, a Global Regulator of Hundreds of Genes, Including Those for Secondary Metabolism, in *Streptomyces venezuelae*

**DOI:** 10.3390/antibiotics14090878

**Published:** 2025-08-30

**Authors:** Marcin Wolański, Małgorzata Płachetka, Volha Naumouskaya, Agnieszka Strzałka, Michał Tracz, Diana Valietova, Jolanta Zakrzewska-Czerwińska

**Affiliations:** Faculty of Biotechnology, University of Wrocław, 50-383 Wrocław, Poland

**Keywords:** secondary metabolite gene cluster/secondary metabolites, transcriptional regulator, gene expression, RNA-seq, ChIP-seq, *Streptomyces venezuelae*

## Abstract

Background: *Streptomyces* bacteria are prolific producers of secondary metabolites (SMs), including many antibiotics. However, most biosynthetic gene clusters (BGCs) remain silent under laboratory conditions. Global transcriptional regulators, such as AdpA, can activate these BGCs, but their roles in secondary metabolism are not fully understood. This study investigates the regulatory function of AdpA in *Streptomyces venezuelae* (AdpA_Sv_), a fast-growing model species and natural chloramphenicol producer that encodes over 30 BGCs. Methods: We applied RNA-seq and ChIP-seq at 12 and 20 h—corresponding to vegetative and aerial hyphae stages—to profile the AdpA_Sv_ regulatory network. Results: AdpA_Sv_ influenced the expression of approximately 3000 genes, including those involved in primary metabolism, quorum sensing, sulfur metabolism, ABC transporters, and all annotated BGCs, and it bound to around 200 genomic sites. Integration of RNA-seq and ChIP-seq data identified a core regulon of 49–91 directly regulated genes, with additional effects likely mediated indirectly via other transcription factors or non-canonical binding sites. Motif analysis confirmed similarity to the canonical *Streptomyces griseus* AdpA-binding sequence, with a novel 5-bp 3′ extension. AdpA_Sv_ directly regulated several SM pathways, including chloramphenicol biosynthesis, potentially alleviating Lsr2-mediated repression. Conclusions: This study defines, for the first time, the direct AdpA regulon in *S. venezuelae* and establishes AdpA_Sv_ as a central regulator of secondary metabolism. Our findings highlight *S. venezuelae* as a promising chassis strain for heterologous expression and suggest strategies for activating silent BGCs in other *Streptomyces* species.

## 1. Introduction

Bacteria of the *Streptomyces* genus are prolific producers of a wide array of specialized metabolites, including anticancer and immunosuppressive drugs, and most naturally derived clinical antibiotics [1,2]. The biosynthesis of these compounds, also called secondary metabolites (SMs), is facilitated by biosynthetic gene clusters (BGCs) frequently comprising several tens of genes. Bioinformatic analyses of the genomes of *Streptomyces* and other members of the Actinobacteria (now Actinomycetota) phylum reveal a high abundance of diverse BGCs, with up to several dozen per strain [3,4]. However, the number of identified SMs remains relatively small, demonstrating an untapped potential for discovering novel bioactive compounds of medical importance in these microorganisms [5,6].

It is assumed that most SMs remain undetected under laboratory conditions due to minimal or absent transcription of the associated BGCs, leading to inadequate biosynthesis of the related compounds [7]. The low transcriptional activity in these “silent” BGCs is frequently attributed to the absence of activating signals, which may include substances from other organisms, stress factors like nutrient scarcity or physical stress, or, in most cases, unidentified triggers [8,9]. Transcriptional regulators (TRs) are crucial in sensing these signals and governing gene expression within secondary metabolite biosynthetic gene clusters (SM-BGCs); however, the functions of most of the TRs remain unstudied [10]. In addition, individual BGCs can be co-regulated by multiple TRs, forming intricate multilevel regulatory networks that enable precise modulation of metabolite biosynthesis in response to environmental stimuli.

TRs involved in SM biosynthesis are typically categorized as either cluster-situated (local) or global regulators [10]. Local regulators are encoded within or adjacent to BGCs and typically regulate the specific expression of associated gene clusters. In contrast, global regulators are not necessarily linked to particular BGCs but instead exert broad transcriptional impacts on the host cells, often influencing multiple SM-BGCs. Due to their role across diverse cellular pathways, leading to pleiotropic effects on host biology, some global regulators are also referred to as central regulators [11,12]. In *Streptomyces*, the most extensively studied SM-related global regulators include AdpA, GlnR, MtrA, and the nucleoid-associated protein (NAP) Lsr2, which are implicated in morphological differentiation, nitrogen metabolism, response to stress conditions, and regulation of secondary metabolism, respectively [13,14,15,16]. Global TRs have emerged as promising tools for the untargeted activation of silent SM-BGCs, in both native and heterologous hosts, and have been shown to effectively trigger the biosynthesis of numerous previously undetectable SMs [17,18,19].

In *Streptomyces*, the biosynthesis of secondary metabolites is often tightly linked to colony differentiation, particularly the transition from vegetative hyphae to aerial hyphae. Antibiotics produced during this stage are thought to serve protective roles, safeguarding nutrients released through the lysis of vegetative mycelium. The transcriptional regulator AdpA plays a central role in coordinating both morphological differentiation and SM biosynthesis. In most *Streptomyces* species—including well-studied model organisms such as *S. griseus*, *S. coelicolor*, and the more recently established model *S. venezuelae*—AdpA is essential for morphological development. Disruption of the *adpA* gene (formerly *bldH*, a member of the “bald” gene group) in these species results in a characteristic “bald” phenotype, marked by the absence of aerial hyphae [20,21,22]. In many *Streptomyces*, developmental defects caused by *adpA* disruption are accompanied by altered biosynthesis of various secondary metabolites, including several antibiotics such as chloramphenicol (Cml), daptomycin, oviedomycin, moenomycin, tunicamycin, and nikkomycin [14,23,24,25]. Beyond its roles in development and SM biosynthesis, AdpA has pleiotropic effects on diverse cellular processes such as nutrient utilization, stress response, and chromosome replication, underscoring its function as a master regulator of *Streptomyces* life cycle [26,27,28].

However, despite its importance, a comprehensive understanding of AdpA’s global regulatory functions, particularly in secondary metabolism, remains limited. To date, only three studies have investigated the AdpA regulon using transcriptomic analysis and chromosomal AdpA-binding site identification, but each was conducted in a different species (*S. coelicolor* or *S. griseus*) [26,29,30]. Moreover, *adpA* expression itself is subject to complex regulatory control at multiple levels, as previously reviewed [31]. Given that AdpA is highly conserved among *Streptomyces* species [32], a detailed investigation of its function in a single model organism could offer broad insights into regulatory networks governing SM biosynthesis throughout the genus—potentially facilitating the activation of cryptic biosynthetic pathways.

In this study, we focus on the recently established fast-growing model *Streptomyces venezuelae*, which harbors over 30 BGCs for secondary metabolites, including chloramphenicol. Unlike other model *Streptomyces* species, *S. venezuelae* is capable of completing the full process of morphological differentiation in liquid media and, consequently, of producing secondary metabolites under these conditions. Due to its rapid and dispersed growth in liquid culture, this species shows strong potential as a chassis strain for the heterologous expression of silent BGCs under scalable fermentation conditions. These features make it an excellent system for studying AdpA’s role in SM biosynthesis.

Here, we present a comprehensive analysis of the regulatory function of the conserved global regulator AdpA in *S. venezuelae* using integrated RNA-seq and ChIP-seq approaches. Our findings reveal that AdpA exerts broad control over gene expression during *S. venezuelae* development, affecting the transcription of several hundred genes involved in diverse biological processes, including all annotated SM-BGCs. Moreover, we demonstrate that AdpA directly regulates the biosynthesis of selected secondary metabolites, including chloramphenicol, and provide new insights into the mechanisms governing expression of the chloramphenicol gene cluster. These results enhance our understanding of AdpA’s regulatory role in SM biosynthesis and further establish AdpA as a promising tool for activating silent secondary metabolite gene clusters.

## 2. Results and Discussion

To define the complete AdpA (vnz_12630) regulon in *S. venezuelae* (hereafter also referred to as AdpA_Sv_), we compared global transcript levels (RNA-seq) between the Sven_ΔadpA/adpA-FLAG strain and the *adpA* deletion mutant (Sven_ΔadpA). The same Sven_ΔadpA/adpA-FLAG strain, along with the wild-type (Sven_WT) (expressing nontagged AdpA from the native locus), was used in ChIP-seq experiments to determine the genome-wide binding profile of AdpA. The construction of both strains was described in our earlier study [14]. RNA-seq and ChIP-seq experiments were conducted under the same cultivation conditions and time points, corresponding to the vegetative (12 h) and aerial mycelium (20 h) growth stages, as reported earlier [14]. These stages coincide with the peaks of AdpA-FLAG protein accumulation during the *S. venezuelae* cell cycle. In our study, we focused particularly on AdpA’s role in regulating the activity of BGCs involved in secondary metabolite production.

### 2.1. AdpASv Exerts a Global Impact on Gene Expression in Streptomyces venezuelae

First, we used RNA-seq to analyze the transcriptional impact of *adpA* gene disruption on global gene expression at two defined developmental stages.

**Figure 1:**
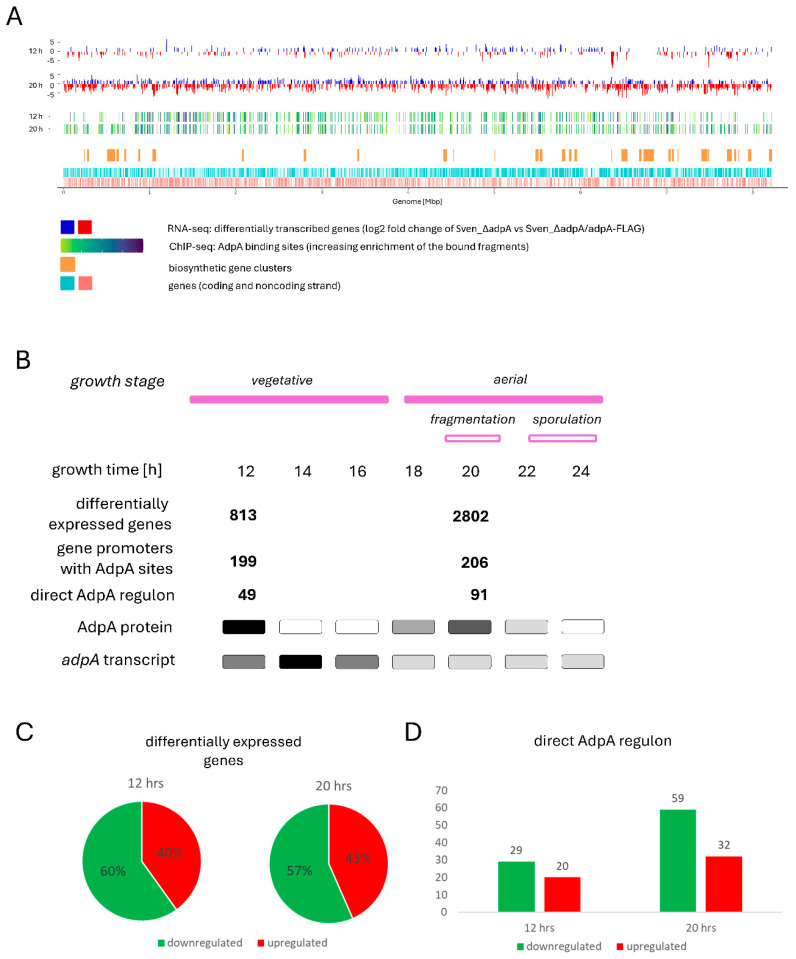
Comprehensive overview of AdpA-dependent gene expression and binding sites in *S. venezuelae*. (**A**) Combined RNA-seq and ChIP-seq data for 12-h and 20-h liquid cultures. The dark blue and red bars in the top panel represent genes whose expression is significantly up- and downregulated in the *adpA_Sv_* mutant (log_2_ fold change). The green to violet bars below indicate the locations of MACS peaks, comprising AdpA_Sv_ binding sites, identified by ChIP-seq. Below that, the orange bars represent the location of biosynthetic gene clusters (see also Table 1). The bottom panel shows the locations of the *S. venezuelae* genes on coding and non-coding strands (light blue and light red) across the chromosome. The R was used to generate the image. (**B**) Comparison of the total number of AdpA_Sv_ binding sites and differentially expressed genes (DEGs) identified (this study) to AdpA_Sv_ transcript and protein levels as measured in a previous study [14]. The approximate increasing abundance of the corresponding measurement is depicted in grayscale from white to black. The direct AdpA regulon is defined as the AdpA-bound gene promoters identified through ChIP-seq analysis within DEGs (see also panel (**D**) and Table S3G). (**C**) The fractions of upregulated and downregulated genes in the *adpA* deletion mutant, as determined by RNA-seq analysis. (**D**) Direct AdpA_Sv_ regulon (Table S3G). For a more intuitive interpretation of AdpA’s transcriptional impact in panels (**C**,**D**), green indicates a positive effect (genes downregulated in the deletion mutant), while red indicates a negative effect (genes upregulated).

RNA-seq analysis comparing the transcriptomes of Sven_ΔadpA and Sven_ΔadpA/adpA-FLAG strains, using standard cut-off parameters (FC ≥ 1.5, with a false discovery rate FDR < 0.05), revealed 813 and 2802 differentially expressed genes (DEGs) at 12 and 20 h, respectively (Figure 1; Table S2A,B). These represent approximately 11% and 39% of the ~7200 chromosomally encoded *S. venezuelae* genes [33].

**Table 1:** Secondary metabolite BGCs in the genome of *S. venezuelae* and their regulation by AdpA.

BGC	BGC Type (Known or Putative Product(s))	Gene Range	No. of Genes	% of Downregulated/Upregulated Genes [12 h] *	% of Downregulated/Upregulated Genes [20 h] *	No. of AdpA Sites **
1	Ectoine (ectoine)	vnz_01060–01105	10	10/0	30/10	1
2	Terpene (geosmin)	vnz_01250–01320	15	20/0	13/33	1
3	PKS-NRPS, NRP-metallophore (venamycin/thiazostatin/watasemycins)	vnz_02200–02530	66	6/0	16/16	0
4	Lanthipeptide-Terpene (chrysomycin, geosmin)	vnz_02580–02675	20	20/5	60/15	2
5	Lanthipeptide (venezuelin)	vnz_02920–02995	16	6/0	6/19	0
6	Indole (rebeccamycin)	vnz_03615–03695	17	6/0	0/24	1
7	NRPS (chloramphenicol)	vnz_04355–04560	42	12/2	55/2	2
8	CDPS (malacidins)	vnz_09090–09185	20	10/0	10/30	1
9	Siderophore (desferrioxamine B)	vnz_12535–12650	23	25/25	8/25	0
10	Lassopeptide (albusnodin)	vnz_15295–15400	22	5/5	5/41	0
11	NRPS (lactonamycin)	vnz_20080–20290	43	0/9	30/2	0
12	Butyrolactone (gaburedins)	vnz_20635–20675	9	22/0	22/0	0
13	Melanin (istamycin, melanin)	vnz_22965–23015	11	18/9	73/0	2
14	Other-Butyrolactone (A-factor)	vnz_25145–25260	24	8/0	33/25	2
15	RiPP-Thiopeptide (BD-12, cutimycin)	vnz_25310–25435	26	15/4	12/4	4
16	PKS (flaviolin, tetrahydroxynaphthalene)	vnz_26470–26620	31	0/0	29/26	0
17	Siderophore (murayaquinone)	vnz_26765–26890	26	4/0	12/42	2
18	Siderophore (kinamycin)	vnz_27055–27160	22	0/5	9/14	2
19	RiPP (ND)	vnz_28840–28880	9	67/0	0/56	0
20	PKS-Butyrolactone (auricin, jadomycin, SCBs)	vnz_29390–29720	67	6/0	30/18	2
21	NAPAA (formicamycins)	vnz_30285–30395	23	4/4	30/26	0
22	NRPS-PKS-RiPP-Thioamide (colibrimycin, esmeralidin)	vnz_30505–30990	98	1/0	36/3	3
23	Terpene (hopene)	vnz_31755–31865	23	0/0	4/4	2
24	Lanthipeptide (SapB)	vnz_31920–32015	19	25/0	20/20	4
25	RiPP (ND)	vnz_32195–32230	8	0/0	63/13	0
26	PKS (spore pigment)	vnz_33355–33670	64	0/3	38/6	1
27	Melanin (melanin)	vnz_33695–33730	8	63/0	0/25	1
28	NRP-metallophore-NRPS (peucechelin, saccharochelins)	vnz_34675–34880	42	76/0	33/5	2
29	Terpene (2-methylisoborneol)	vnz_34995–35080	18	0/6	67/6	0
30	hydrogen–cyanide (aborycin)	vnz_35165–35215	11	0/0	36/0	0
31	PKS (alkylresorcinol)	vnz_35605–35770	34	6/0	29/6	0
32	Terpene-NRPS (ND)	vnz_36585–36745	33	0/3	27/12	1

Biosynthetic gene clusters (BGCs) by antiSMASH (v7.1.0) and the reference genome NZ_CP018074.1 of *S. venezuelae* NRRL B-65442. BGC region numbering and gene cluster boundaries are based on antiSMASH results as of 2025-04-16. Abbreviations: CDPS—tRNA-dependent cyclodipeptide synthase; NAPAA—non-α poly-amino acids (e.g., ε-polylysine); ND—not determined; NRPS—nonribosomal peptide synthase; PKS—polyketide synthase; RiPP—ribosomally synthesized and posttranslationally modified peptide. * % of genes comprising the corresponding BGC listed in Table S5. ** total number of FIMO identified AdpA binding motifs in the BGC based on Table S5.

**Figure 2:**
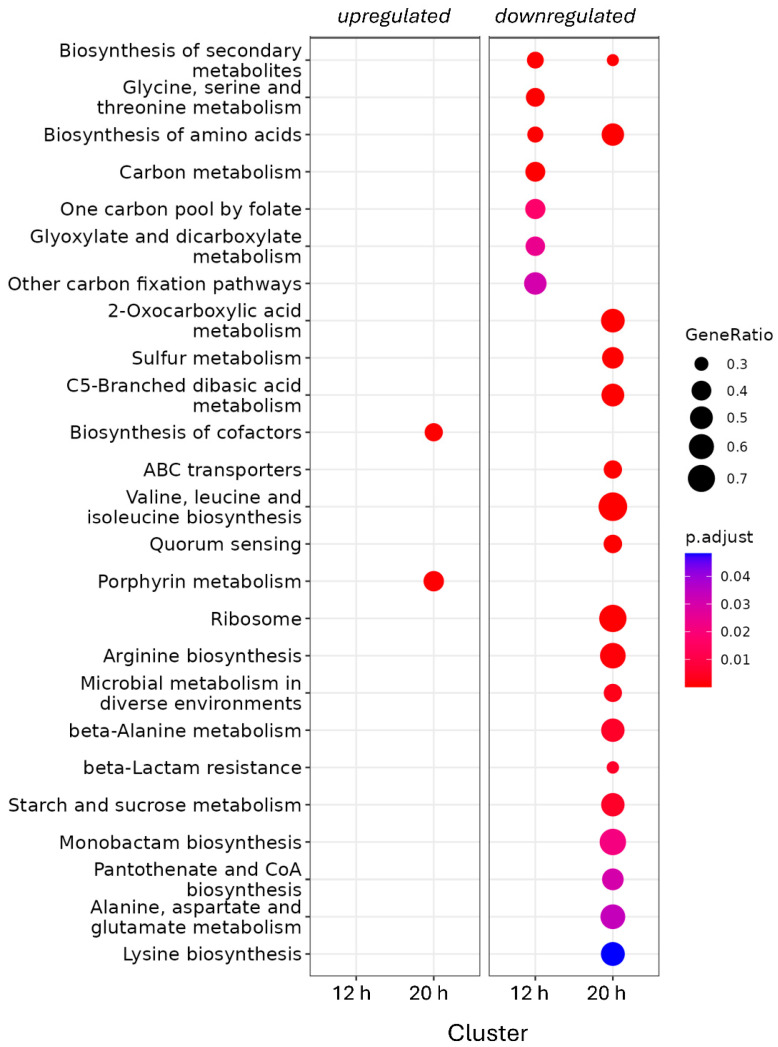
Metabolic pathways affected by AdpA_Sv_. The graph displays enriched metabolic pathways that are either activated (**left**) or suppressed (**right**) in the Δ*adpA* strain compared to the wild-type strain at 12 and 20-h time points, based on RNA-seq data. The DEGs were grouped into corresponding pathways according to the KEGG database. The GeneRatio parameter shows the proportion of pathway-associated genes that are significantly changed. Statistical significance, expressed as adjusted *p*-value, is reflected by color intensity: red indicates high significance (low adjusted *p*-value), while blue indicates low significance (high adjusted *p*-value). Gene grouping analysis was performed in R using the ClusterProfiler package.

At both time points, the majority of DEGs were downregulated in the *adpA* deletion mutant —487 out of 813 genes (60%) at 12 h and 1585 out of 2802 genes (57%) at 20 h—suggesting that AdpA_Sv_ primarily functions as a transcriptional activator (Figure 1C). Interestingly, only 347 genes were shared between the sets of DEGs at both time points (Table S2C), representing approximately 43% and 12% of the regulated genes at 12 and 20 h, respectively. The distribution of gene expression changes based on KEGG pathway analysis revealed functional divergence between the two time points (Figure 2 and Figure S1). The differentially expressed genes were predominantly involved in primary metabolism, with pathways such as carbon metabolism and glyoxylate and dicarboxylate metabolism mainly affected at 12 h; sulfur metabolism, quorum sensing, ABC transporters, and biosynthesis of cofactors and porphyrins affected at 20 h; and secondary metabolite biosynthesis and amino acid biosynthesis impacted at both time points.

Enrichment distribution analysis revealed more pronounced gene expression changes at 20 h compared to 12 h, with a higher proportion of pathway-associated genes (GenRatio) affected by AdpA_Sv_ at the later time point (Figure 2). This suggests that AdpA_Sv_ plays a pivotal role in coordinating gene expression in a growth stage-dependent manner. Interestingly, the top twenty differentially expressed genes (DEGs), based on fold change at both time points, are primarily involved in core metabolic processes according to KEGG annotation. These include amino acid biosynthesis and degradation, sulfur metabolism, fatty acid biosynthesis, as well as ABC transporters and oxidative phosphorylation (Figure S2; Table S2D,E).

Our findings indicate that AdpA_Sv_ influences the transcription of a substantial portion of the *S. venezuelae* genome—11% and 39% of genes at the two respective developmental stages—supporting its role as a global regulator. The substantial increase in AdpA_Sv_-regulated genes at 20 h is consistent with its established role in controlling genes involved in *Streptomyces* morphological differentiation [14,21,28,34], and suggests that the AdpA_Sv_ regulon undergoes a significant, growth-stage-dependent shift. Notably, AdpA_Sv_ was found to positively correlate with the expression of several key developmental genes [35,36], including: *chpC*, *chpD*, *chpF*, *rdlB*, encoding chaplins and rodlins, which act together in aerial hyphae emergence and formation of the hydrophobic spore surface [37,38,39]; *whiE* cluster (*whiE*-ORFII to ORFVII), which is transcriptionally active during sporulation septum formation and responsible for polyketide spore pigment production [40]; *whiD*, whose protein product exact function remains unclear but which is involved in late stages of sporulation [41,42]; *sti1*, encoding a secreted trypsin-like protease inhibitor likely involved in regulating endogenous and environmental protease activity during development [43,44], and *sgmA*, encoding an extracellular protease likely involved in development (see also further discussion) [45] (Table S2F).

Notably, as shown above, approximately two-thirds of the DEGs were positively regulated by AdpA_Sv_, consistent with findings in *S. lividans*, where ~64% of AdpA (AdpA_Sl_) target genes were also reported to be activated [29]. However, the smaller number of AdpA_Sl_-dependent genes reported in that study (~300) may reflect slight differences in the growth stages analyzed and AdpA expression levels. While the previous work focused on the early (likely vegetative) stage, our analysis encompassed both the vegetative and early aerial hyphae (fragmentation) stages, during which AdpA levels peak.

Interestingly, our data show that the regulatory impact of AdpA_Sv_, measured by the number of affected genes, is higher at the later time point (813 at 12 h versus 2802 at 20 h), despite *adpA* transcript and AdpA protein levels peaking at earlier stages, as reported here (Figure S3) and in our previous study [14]. This discrepancy suggests that AdpA_Sv_ activity may be modulated posttranslationally, through interactions with specific ligand(s), or by changes in DNA accessibility resulting from chromosomal rearrangements. One plausible mechanism is sulfane sulfur modification of a conserved cysteine residue, recently reported for AdpA in *S. coelicolor* [46]. However, this mechanism has only been studied scarcely in *Streptomyces*, and the specific conditions under which it occurs remain unclear. Previous studies have shown that extensive chromosomal rearrangements occurring at the onset of differentiation in various *Streptomyces* species can cause substantial changes in global transcription and chromosome replication [47,48]. These processes have been proposed to influence the activity of other transcriptional regulators, including those involved in secondary metabolism, and could similarly affect AdpA_Sv_ binding activity (see also discussion in later sections).

We further hypothesize that AdpA_Sv_ activity may be modulated by an as-yet unidentified redox-related mechanism, potentially linking its regulatory function to oxygen-limited conditions. This hypothesis is supported by the pronounced shift in the set of DEGs in the *adpA* mutant strain under oxygen-depleted conditions, as revealed by our preliminary analyses (Table S2G). Additionally, the observed differences in gene expression between the two time points may mostly result from indirect regulation via other transcriptional regulators and changes in chromosome topology, which are addressed in subsequent sections.

### 2.2. AdpA_Sv_ Directly Binds About 200 Gene Promoters Across the Genome

To determine which of the observed AdpA-dependent changes in gene expression result from direct binding of AdpA to gene promoters, we performed ChIP-seq. The analysis was conducted at the same time points as the RNA-seq experiment, using the wild-type strain (Sven_WT) as a non-binding control.

Initial analysis of enriched DNA fragments (MACS peaks) revealed 572 and 655 AdpA-bound regions (mean lengths of 1050 bp and 944 bp) at 12 and 20 h, respectively (Figure 1A, Figures S4 and S5; Table S3A,B). Remarkably, around 70% of these regions were shared (overlap of at least 50 nucleotides between fragments) between the two time points.

**Figure 3:**
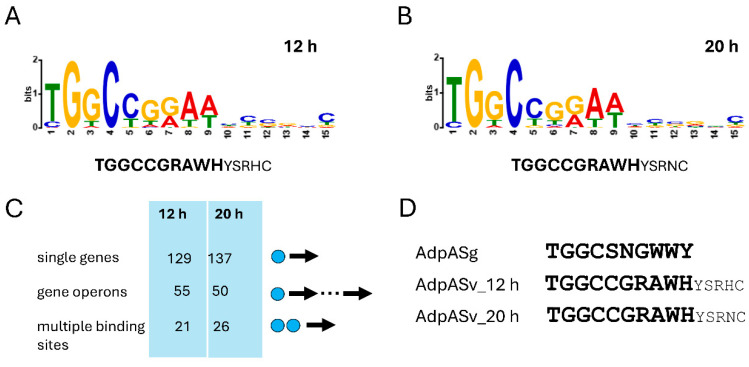
AdpA_Sv_-binding consensus sequence. (**A**,**B**) sequence logos of AdpA-binding motifs identified by MEME-ChIP from ChIP-seq data of *S. venezuelae* liquid cultures harvested at 12 and 20 h, respectively. The corresponding consensus sequences are shown below each logo. (**C**) Distribution of AdpA-binding motifs in gene promoters, categorized by gene organization and the number of binding sites: single genes—motifs located in promoters of individual genes or shared promoters of divergently oriented gene pairs; gene operons—motifs located in promoters upstream of operons; multiple binding sites—at least two AdpA-binding motifs present in promoters of single genes or shared promoters of divergent gene pairs. Arrows represent genes, and blue circles represent AdpA-binding motifs. (**D**) Comparison of AdpA-binding motifs identified in *S. venezuelae* (AdpA_Sv_; this study) and *S. grisues* (AdpA_Sg_; [50]). Nucleotide codes follow the IUPAC ambiguity code: S = G or C; W = A or T; Y = T or C; R = G or A; H = A or C or T; N = any nucleotide.

To identify an AdpA_Sv_ binding motif, we analyzed the MACS peak sequences using the MEME-ChIP tool [49]. Given that previous studies showed that AdpA homologs exhibit relaxed binding specificity, we applied stringent criteria (fold change ≥ 1.75; see Section 3) to increase the reliability of motif identification. This analysis revealed two nearly identical 15-bp sequence motifs: 5′-TGGCCGRAWH(YSRHC)-3′ and 5′-TGGCCGRAWH(YSRNC)-3′, corresponding to the 12-h and 20-h samples, respectively (Figure 3). Both motifs share a conserved 10-bp core sequence (5′-TGGCCGRAWH-3′), which aligns well with the canonical AdpA binding motif 5′-TGGCSNGWWY-3′ previously described for the *Streptomyces griseus* homolog AdpA_Sg_ [50]. Interestingly, the 3′ ends of the motifs contained a less conserved, 5-bp stretch (YSRHC and YSRNC, respectively) showing a single nucleotide difference (H vs. N).

We next used the FIMO output from MEME (threshold *p*-value < 1 × 10^−4^), which defines the exact positions of identified motifs within MACS peaks, to map AdpA-binding sites across the *S. venezuelae* genome. This analysis identified a total number of 238 and 245 AdpA motifs at 12 and 20 h, respectively, of which 175 and 182 were within promoter regions (+350 to −50 bp relative to the start codon) of 199 and 206 genes at 12 and 20 h, respectively (Figure 1B; Table S3C,D). The majority of the target genes (161) were shared between the two time points (Table S3E). In approximately two-thirds of all cases, AdpA-binding motifs were located within promoters of single genes or shared promoters of divergently oriented gene pairs. Additionally, AdpA motifs were identified in promoters of genes forming putative operons—55 and 50 cases at 12 and 20 h, respectively (Table S3E)—suggesting that genes within these operons may undergo direct regulation by AdpA (Figure 3C). Given that AdpA, as a global regulator, presumably interacts with degenerate binding sites, we further investigated this aspect by scanning the genome for relaxed AdpA binding motifs (*p*-value ≤ 1.04 × 10^−4^) using the FIMO tool from the MEME suite. This analysis identified 5197 potential binding sites, of which 1997 were located in promoter regions, corresponding to 1624 potentially regulated genes (Table S3F; see also next section).

Previous and current studies have demonstrated substantial variation in the estimated number of genes with AdpA-binding sites in their promoter regions across different *Streptomyces* species, ranging from approximately 50–160 in *S. coelicolor* [34,51], ~320 in *S. lividans* [29], ~700–900 in *S. griseus* [26], to ~200 in *S. venezuelae* (this study). However, it should be noted that the first three datasets (for *S. coelicolor* and *S. lividans*) were primarily based on in silico predictions, while later studies (on *S. grisues* and *S. venezuelae*) combined ChIP-seq with bioinformatic motif discovery. In our analysis, we applied a stringent threshold (fold change ≥ 1.75), which likely reduced the total number of detected sites but enriched for highly conserved motifs. Notably, our study and the original analysis in *S. griseus [50]* identified highly similar consensus motifs, supporting the robustness of this core binding sequence.

We reason that these discrepancies likely reflect both biological divergence among *Streptomyces* species (e.g., differences in growth rate or the ability to differentiate in various media) and experimental variation (e.g., cultivation conditions, media composition), including methodological differences. Importantly, they are not related to genome size, which is similar across these species.

Our study revealed that the core AdpA-binding motif in *S. venezuelae* (5′-TGGCCGRAWH-3′) is nearly identical to that in *S. grisues* (5′-TGGCSNGWWY-3′), supporting previous findings that highlight the importance of conserved G and C residues at positions 2 and 4 for AdpA binding specificity [50,52]. Recognition of an identical core motif is likely due to the 100% amino acid sequence identity in the DNA-binding domains (DBDs) of AdpA_Sg_ and AdpA_Sv_. Given the high conservation of DBDs among AdpA homologs [32], it is plausible that these homologs recognize the same nucleotide sequences.

In silico motif searches revealed a 5-bp extension at the 3′ end of the AdpA binding motif. Although the functional relevance of this 3′ extension sequence remains unclear, crystallographic studies [52] suggest that flanking nucleotides may stabilize AdpA dimers or oligomers, particularly when binding occurs at clustered sites. This implies that the 3′-flanking region could structurally contribute to the formation of the AdpA-DNA complex. We hypothesize that, while its precise role is yet to be determined, it may influence the binding specificity of AdpA oligomers to adjacent relaxed binding sites, as discussed below.

Our in silico screening further demonstrated that a total of 1624 genes were associated with the presence of relaxed binding sites in their promoter regions. AdpA’s low DNA-binding specificity enables interaction with numerous degenerate chromosomal sites, providing functional plasticity for broad yet fine-tuned regulation of genes, including those involved in secondary metabolite production, in response to diverse environmental signals and physiological cues (Browning & Busby, 2004; Higo et al., 2012). It has also been shown that intracellular dynamics, such as chromosome remodeling—including changes in DNA topology and chromosome structure—during bacterial development may facilitate global transcriptional reprogramming by altering the accessibility of transcription regulator binding sites [48,53]. Moreover, AdpA’s ability to bind relaxed motifs may contribute to regulatory redundancy, whereby multiple regulators control overlapping gene sets, enhancing both the robustness of essential metabolic pathways and the evolutionary flexibility of regulatory networks [54,55]. It has been proposed that AdpA’s low DNA-binding specificity may increase the likelihood of its binding sites appearing in the regulatory regions of newly acquired genes, including foreign biosynthetic genes involved in secondary metabolites production [26]. However, determining the functional relevance of such relaxed sites remains a significant challenge. Nonetheless, our results suggest a broader and more complex AdpA regulatory network, as further illustrated by its confirmed binding within the chloramphenicol biosynthetic gene cluster (see below).

### 2.3. AdpA Directly Regulates a Limited Set of Genes Including Developmental Genes but Likely Exerts Most of Its Transcriptional Effects Through Other Regulators

To identify genes directly regulated by AdpA_Sv_, we integrated RNA-seq data with stringent ChIP-seq analysis. For AdpA-binding sites located in the promoter regions of putative operons (see discussion above), only the genes closest to the binding site in the operon were counted.

This approach revealed a relatively small subset of 49 and 91 genes at the 12- and 20-h time points, respectively, that were both differentially expressed and associated with AdpA-binding motifs in their promoter regions, thereby defining a putative direct AdpA regulon (Figure 1D; Table S3G). These targets included genes encoding transcriptional regulators, proteases, and enzymes involved in primary metabolism. However, only 20 direct targets were shared between the two time points. Notably, 16 of these directly regulated genes are located within secondary metabolite biosynthetic gene clusters (SM-BGCs) (Tables S3G and S5).

AdpA predominantly functioned as a transcriptional activator, with a positive correlation between AdpA binding and elevated gene expression observed in 59% and 65% of cases at 12 and 20 h, respectively (Table S3G). Notably, the relaxed binding site approach (see previous section) revealed that out of 1624 genes associated with relaxed AdpA binding sites in their promoters, 189 and 663 genes were differentially expressed in the *adpA* deletion mutant at the 12- and 20-h time points, respectively. This suggests that these genes could also be potential direct targets of AdpA regulation (Table S3F).

The strictly defined regulon (excluding downstream operon genes and relaxed sites) accounted for ~6% and ~3% of the total DEGs in the *adpA* mutant, at 12 and 20 h, respectively. These proportions are consistent with previous studies of some of the global transcriptional regulators (e.g., RsaL ~4%, MexT ~6% and PchR ~8% as reported in [56]. Our analysis revealed that the composition of the direct AdpA regulon changes substantially across developmental stages—49 genes at 12 h versus 91 at 20 h—indicating a dynamic, growth-stage-dependent shift in the AdpA regulon, consistent with AdpA’s established roles in development and secondary metabolism.

Among the direct AdpA targets are two developmental genes: *whiB* (vnz_13645), which is negatively regulated, and *sgmA* (vnz_25255), which is positively regulated. In *S. coelicolor*, the transcriptional regulator WhiB (SCO3034) coordinates sporulation septation in cooperation with WhiA, collectively controlling hundreds of genes, including those essential for sporulation [57,58]. SgmA (SCO5447) encodes an extracellular metalloprotease thought to contribute to aerial mycelium formation. Although *sgmA* deletion had no clear phenotype in *S. coelicolor*, this was attributed to redundancy with the adjacent homologue SCO5446 [45]. Interestingly, *S. venezuelae* carries only a single copy of *sgmA*, and its AdpA-dependent activation during late growth stages suggests a potential role in differentiation—though this function remains uncharacterized in this species.

To explain the broad transcriptional impact of AdpA on gene expression, we investigated the possibility of indirect regulation via other transcriptional regulators. Specifically, we analyzed the influence of AdpA on the expression of a combined set of 49 regulatory genes referenced in the previous studies, including known global regulators, transcription factors associated with secondary metabolism, and those cataloged in the LogoMotif database [13,59,60].

Depending on the time point, 3 and 17 out of these regulators were differentially expressed in the *adpA* deletion mutant at 12 and 20 h, respectively (Table S4A). Notably, these included several key global regulators: MtrA (vnz_13525), which is involved in chromosome replication, cell division, development, and secondary metabolism [16,61,62]; Rex (vnz_15640), a redox-sensing regulator involved in anaerobic and secondary metabolism [63,64,65]; WblA (vnz_16495), a global negative regulator of secondary metabolism and a positive regulator of sporulation [66,67], and WhiAB (vnz_07750, vnz_13645), which cooperatively regulate antibiotic resistance, pathogenesis, and development [57,68]. The differential expression of these major regulators, along with significant changes in their corresponding regulons—as exemplified by the Rex and WhiAB regulons (Table S4B)—in the *adpA* mutant supports the idea that AdpA exerts much of its global regulatory influence indirectly, by modulating other transcriptional networks. Moreover, recent studies have shown that global chromosome topology and spatial rearrangements of the chromosome play important roles in the regulation of transcriptional activity in *Streptomyces*, including the expression of numerous BGCs. These structural dynamics are generally thought to influence the accessibility of DNA to transcriptional regulators, thereby modulating gene expression on a broad scale [47,48,69,70]. Notably, certain global regulators, such as Lsr2, a NAP, are believed to induce topological changes in the chromosome [71,72], thereby further amplifying their transcriptional regulatory impact.

### 2.4. AdpA_Sv_ Directly Affects the Majority of SM-BGCs and Associated Metabolite Biosynthesis

Gene expression analysis revealed that AdpA_Sv_ influences multiple metabolic pathways, including the biosynthesis of secondary metabolites. To assess AdpA_Sv_ global impact on specialized metabolism, we analyzed the distribution of AdpA-binding sites and associated changes in gene expression across SM-BGCs identified in the *S. venezuelae* genome using the recent release of antiSMASH.

RNA-seq analysis showed that all 32 SM-BGCs exhibited altered transcriptional profiles in the *adpA* deletion mutant Sven_ΔadpA compared to the Sven_ΔadpA/adpA-FLAG. However, ChIP-seq-based analysis detected AdpA-binding motifs in only 17 of these clusters (Table 1 and Table S5), suggesting that AdpA directly regulates around half of the SM-BGCs and exerts indirect control over the rest.

The regulatory impact of AdpA was more pronounced at the 20-h time point, as reflected by both the number of affected clusters (29 at 12 h vs. 32 at 20 h) and a higher average proportion of DEGs within clusters (38% vs. 16%) (Table 1).

Notably, at both time points, *adpA* deletion led predominantly to downregulation of gene expression. Approximately two-thirds of all DEGs in the BGCs were downregulated, indicating that AdpA primarily acts as a positive regulator (Table S5). This trend was also observed at the level of individual clusters: at 12 h, 20 out of 29 clusters exhibited a higher proportion of downregulated than upregulated genes in the deletion strain, while 5 clusters showed the opposite pattern and 4 showed equal proportions (Table 1). A similar distribution was observed at 20 h, with 19 out of 32 clusters showing predominant downregulation, 9 showing upregulation, and 4 exhibiting balanced expression changes.

A strong transcriptional response—defined as differential expression of more than 50% of genes within a BGC—was observed in 3 clusters at 12 h and 10 clusters at 20 h (Table 1). Of these, 2 (12 h) and 5 (20 h) contained AdpA-binding motifs, supporting a potential direct regulatory role. Additionally, the expression of local regulatory genes within SM-BGCs was affected in 19 of the 32 clusters at one or both time points (Table S5), suggesting that AdpA may also exert indirect effects through modulation of cluster-specific regulators.

To assess the impact of AdpA on SM-BGCs, we examined the production of metabolites corresponding to several selected clusters. These SM-BGCs were chosen based on whether they encode well-characterized compounds, the presence of AdpA-binding sites, or a strong transcriptional response. These included BGCs encoding Cml (BGC 7), desferrioxamine (BGC 9), melanin (BGC 13), A-factor (BGC 14), kinamycin (BGC 18), SapB (BGC 24), peucechelin (BGC 28), and 2-methylisoborneol (BGC 29) (Table 1), as well as coproporphyrin, which is not associated with a specific gene cluster. Since our preliminary studies showed that coproporphyrin production occurs only under low shaking conditions (140 rpm), we conducted analyses under both standard (220 rpm) and low shaking conditions.

**Figure 4:**
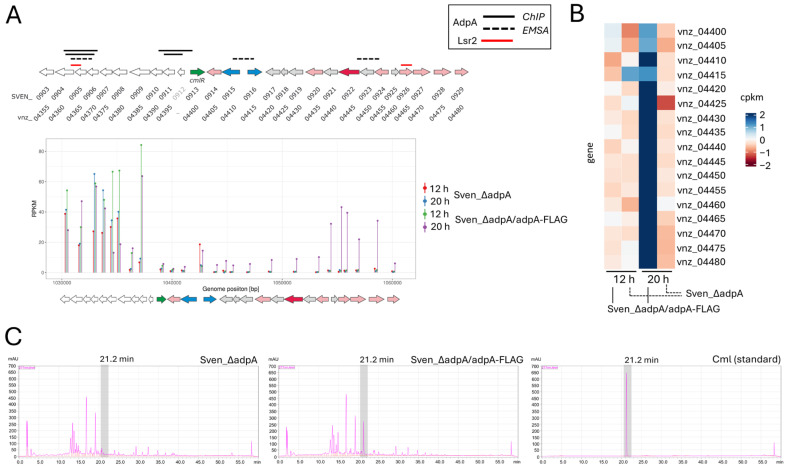
Role of AdpA in the regulation of chloramphenicol biosynthesis in *Streptomyces venezuelae.* (**A**) Genomic organization of the chloramphenicol biosynthetic gene cluster (Cml-BGC) and flanking genes (SVEN_0903–0929) (top panel), and corresponding gene expression levels across this region, expressed as RPKM (bottom panel). Horizontal bars in the top panel indicate AdpA binding regions identified in this study using ChIP-seq and EMSA (black bars; see Table S3A,B and Figure S6). From top to bottom, the solid bars represent fragments identified by ChIP-seq at 12 and 20 h, respectively (MACS peaks: FLAG12_WT12_peak_39, FLAG12_WT12_peak_40, FLAG20_WT20_peak_96, and FLAG20_WT20_peak_97; Table S3A,B). Lsr2 binding regions, based on previous work by Zhang et al. [72], are indicated with a red bar. Gene coloring in the cluster follows antiSMASH convetions: green—regulatory genes, blue—transport-related genes, red—core biosynthetic genes, light red—additional biosynthetic genes, grey—other genes. (**B**) Heatmap of expression levels (in CPKM) of core biosynthetic genes involved in chloramphenicol production (SVEN_0913–0929). (**C**) HPLC chromatograms showing chloramphenicol production in extracts from liquid cultures of *S. venezuelae* strains: the AdpA deletion mutant (Sven_ΔadpA) and the strain expressing AdpA-FLAG (Sven_ΔadpA/adpA-FLAG). Chloramphenicol elutes at approximately 21.2 min (highlighted by a vertical grey bar).

The results revealed a positive effect of AdpA on the accumulation of Cml and melanin under both conditions, desferrioxamine B under standard shaking (220 rpm), and metalated coproporphyrin only under low shaking (140 rpm) (Figure S7 and Table S6). Under the tested conditions, no production or significant differences were observed for the other selected metabolites in extracts from the Sven_ΔadpA strain compared to the Sven_ΔadpA/adpA-FLAG strain. Further, chemical analyses (HPLC, LC-MS) revealed approximately 30-fold decrease in Cml biosynthesis in the *adpA* deletion strain under standard conditions (Figure 4 and Figure S8; Table S6), with production levels of ~510 µg/L in the production medium in the Sven_ΔadpA/adpA-FLAG strain and undetectable levels (below the linear detection range) in the Sven_ΔadpA. The production levels of melanin, desferrioxamine, and coproporphyrin were also significantly affected, with changes ranging from several to several dozen times depending on the cultivation conditions (Table S6).

Our transcriptional analysis demonstrates that the chloramphenicol gene cluster (Cml-BGC, BGC 7 in Table 1) [73], previously proposed as an AdpA target [14], was one of the most strongly affected clusters. In the Δ*adpA* mutant, 12% of its genes were downregulated at 12 h, rising to 55% at 20 h—while only ~2% were upregulated at either time point. Importantly, by 20 h, all essential biosynthetic, resistance, and regulatory genes within the Cml-BGC (vnz_04400–04480/SVEN_0913–0929, corresponding to “vnz” and “SVEN” gene annotation) exhibited AdpA-dependent expression (Tables S5 and S7).

We speculate that the observed transcriptional effects are at least partially attributed to AdpA binding sites identified by our ChIP-seq analysis in proximity to the Cml-BGC: one upstream of vnz_04360 (SVEN_0904), flanking the cluster, and another adjacent to *cmlR* (vnz_04400/SVEN_0913). Notably, the upstream site overlaps with an Lsr2-binding region. Lsr2 represses chloramphenicol biosynthesis by polymerizing along the DNA and bridging the upstream site with vnz_04460–04465, thereby silencing transcription [72]. Our findings suggest that AdpA competes with Lsr2 for binding at this shared region, disrupting Lsr2-dependent DNA loops and relieving repression.

Although the AdpA-binding motif adjacent to *cmlR* lies approximately 517 bp upstream of the gene, ChIP-seq data alone cannot fully explain the direct activation of this gene observed in RNA-seq. However, in vitro assays demonstrated that AdpA binds not only to the region upstream of vnz_04360, but also to two additional regions within the cluster: the intergenic promoter vnz_04410–04415 (SVEN_0915–0916) and a site spanning vnz_04445–04450 (SVEN_0922–SVEN_0923), both implicated in chloramphenicol resistance and biosynthesis, respectively (Figure S6, Table S6). These results support the previously discussed concept that AdpA_Sv_ can interact with relaxed binding motifs, targeting at least one key regulatory element within the Cml-BGC.

Our study demonstrated that AdpA_Sv_ positively influences the biosynthesis of desferrioxamine B and coproporphyrin, both of which are associated with iron metabolism and are typically produced under iron-limiting conditions [74,75,76]. Interestingly, antiSMASH analysis indicates that *adpA* may be part of the desferrioxamine B gene cluster (Table S5), suggesting a possible role for AdpA_Sv_ as a cluster-situated regulator. In both cases, AdpA was associated with downregulation of gene expression within the corresponding clusters. This discrepancy may reflect differences in cultivation conditions: metabolite production was assessed in common secondary metabolite production medium, GYM, whereas ChIP-seq and RNA-seq analyses were conducted in standard MYM medium.

Notably, Cml has also been reported to bind iron [77], raising the possibility that, in addition to its antibiotic activity, it may contribute to iron sequestration, potentially limiting iron availability to competing microorganisms when produced by *S. venezuelae*.

Melanins are multifunctional pigments in *Streptomyces*, known for their UV-absorbing properties, metal ion chelation, antimicrobial activity, redox behavior, and free radical scavenging [78]. In our study, AdpA showed a positive correlation with the expression of the melanin gene cluster and bound to two sites within this cluster, providing strong evidence for its direct regulatory role. However, the specific function of melanin in *S. venezuelae* was not further investigated.

Our analysis revealed key insights into AdpA’s regulation of multiple BGCs, but further proteomic studies are needed to deepen this understanding. Integrating transcriptomic and proteomic data would provide a complementary view of secondary metabolism and other cellular processes, enhancing the dissection of biosynthetic pathways and strategies for activating silent gene clusters.

In summary, our secondary metabolite production data, together with ChIP-seq and RNA-seq analyses, support a broad regulatory role for AdpA in the biosynthesis of diverse specialized metabolites and highlight its potential as a valuable tool for activating secondary metabolism.

## 3. Materials and Methods

### 3.1. Strains and Growth Conditions

Bacterial strains used in this study are listed in Table S1. The growth conditions for the ChIP-seq and RNA-seq experiments are described below in the corresponding sections.

### 3.2. Chromatin Immunoprecipitation-Sequencing and Bioinformatics Analysis

A ChIP-seq was performed according to the previously published protocol [61], with the modifications to the growth conditions described in the later report [14]. Briefly, the liquid maltose–yeast extract–malt extract (MYM) medium (maltose 4 g/L, yeast extract 4 g/L, malt extract 10 g/L; pH 7.3) supplemented with trace elements was inoculated to a final optical density (OD) of 0.001 with the spores of *S. venezuelae* strains (Sven_WT—wild-type, control sample, and Sven_ΔadpA/adpA-FLAG—*adpA* deletion mutant complemented with *adpA-FLAG* under *adpA* native promoter) [14], and incubated for 12 and 20 h at 30 °C with shaking (220 rpm). The cultures were prepared in two independent biological repetitions per strain using standard 250-mL flasks (flat bottom, w/o baffles) containing 50 mL of the medium. The subsequent steps, including cross-linking, DNA-protein complexes isolation using Anti-FLAG M2 Magnetic Beads (Sigma-Aldrich, St. Louis, MO, USA; M8823), and the downstream procedures, were performed as described in the previous study [14]. A corresponding “input” DNA was prepared for each immunoprecipitation sample by taking a small fraction of the sonicated sample, followed by DNA precipitation. For all samples, the purified DNAs were subject to library preparation and Illumina sequencing at the Laboratory of Molecular Neurobiology Centre in Nencki Institute of Experimental Biology (Warszawa, Poland), as described earlier [79].

ChIP-seq reads were mapped to the *S. venezuelae* genome using NZ_CP018074.1 *bowtie2* (version 2.5.2) [80] and processed with samtools (version 1.19.2) [81]. The MACS2 software (version 3.0.2) [82] was employed for peak calling with a nomodel setting. Peaks were considered significant if the q-value was below 0.05 and the fold change was above 1.5.

### 3.3. Identification of Regulatory Protein Binding Motif

The enrichment of the AdpA binding motif in ChIP-seq fragments was analyzed using the online MEME-ChIP tool (version 5.5.7, MEME Suite) [49,83]. Before the analysis, the peaks derived from MACS2 software (MACS peaks) were processed using an R script to center them to the absolute summit (abs_summit), trimmed to 100 bp upstream and downstream relative to a summit position (resulting in 201-bp fragments), and filtered to include only peaks with a fold enrichment ≥ 1.75, to maximise the accuracy of prediction. The subsequent motif discovery was performed by MEME-ChIP analysis in classic discovery and enrichment mode, using the tool’s default parameters except for the expected motif site distribution option, which was set to “Any number of repetitions”. The identified AdpA binding motifs were mapped onto the chromosome using an R script, based on the motif coordinates automatically generated by FIMO (Find Individual Motif Occurrences) [84], as part of the MEME-ChIP analysis. An additional search for the occurrence of relaxed AdpA motifs (*p*-value ≤ 1.04 × 10^−4^) in the genome sequence was performed using the FIMO tool (version 5.5.8) (NZ_CP018074.fa). 

The interactions with selected DNA fragments comprising AdpA-binding motifs were confirmed using electrophoretic mobility shift assays (EMSA) following previously published protocols [14,85], see the Supplementary Information (SI).

### 3.4. RNA-Sequencing and Bioinformatics Analysis

The RNA-seq was performed in three biological replicates using Sven_ΔadpA/adpA-FLAG and Sven_ΔadpA strains grown under the ChIP-seq protocol conditions (see above). For details on the growth conditions of oxygen-depleted cultures, see the Supplementary Information. The sampling of *S. venezuelae* cultures followed by RNA isolation was performed as previously reported [14]. Briefly, at the time of collection (12 and 20 h), the culture suspensions were mixed with the stop solution (95% ethanol and 5% water-saturated phenol, pH 4.5–5; CarlRoth, Karlsruhe, Germany; A980.1) and immediately spun down. After discarding the supernatants, the cell pellets were stored at −80 °C for further use. RNA was isolated using TRI Reagent Solution (ThermoFisher, Waltham, MA, USA; AM9738) and zirconia/silica beads (Carl Roth, Karlsruhe, Germany; N033.1), with cell disruption performed using a FastPrep-24 homogenizer (MP Biomedical, Irvine, CA, USA). The aqueous phase was then purified using the Total RNA Mini Kit (A&A Biotechnology, Gdańsk, Poland; 031-100), following the manufacturer’s instructions. The concentration and quality of the isolated RNAs were examined using a NanoDrop ND-1000 spectrophotometer, followed by “bleach-gel” electrophoresis [86]. To remove the traces of co-purified genomic DNA, the RNA samples (6 µg) were treated with DNase I (6 U per sample, ThermoFisher Scientific, Waltham, MA, USA; EN0521) according to the manufacturer’s recommendations, followed by column purification (ThermoFisher Scientific, Waltham, MA, USA; K0842). The concentration and integrity of the DNase I-treated RNAs were examined again as in the previous step. The 1.8–4 µg of total RNA samples, in the final volume of 20 mL, were submitted to Genewiz (Leipzig, Germany) for standard RNA-seq service including pre-processing (additional DNase I treatment and rRNA depletion), library preparation, and sequencing (Illumina HiSeq 2 × 150 bp, single index, pair-ended reads). Only the RNA samples that, after in situ QC, exhibited RIN values > 6.0 (TapeStation Analysis Software, version 3.2) were subjected to sequencing.

RNA-seq reads were mapped to the *S. venezuelae* genome NZ_CP018074.1 using *bowtie2* (version 2.5.2) [80] and processed with samtools (version 1.19.2) [81]. On average, 10^7^ reads were mapped successfully to the *S. venezuelae* genome. Differential analysis was performed using R packages Rsubread (version 2.10) and edgeR (version 3.38) [87,88] following a protocol described in [89]. After removal of genes with low expression (less than 10 reads in all libraries), the gene count matrix was normalized using edgeR’s calcNormFactors function, and a quasi-likelihood negative binomial was fitted to the data with the glmQLFit function. Differential expression was tested using the glmTtreat function with a 1.5-fold change threshold. Genes were considered to be differentially expressed if the false discovery rate (FDR) was below a 0.05 threshold and the log fold change (logFC) ≥ 1.5. For data visualization, the ggplot2 (version 3.3.6) [90] R package was used. For gene grouping analysis, the ClusterProfiler (version 4.16.0) [91] R package was used. Function gseKEGG with pvalueCutoff = 0.05 was used to determine which pathways were enriched in the RNA-seq data. Only pathways with a q-value < 0.05 were used in further analysis.

### 3.5. Secondary Metabolite Extraction

The extraction of metabolites from *S. venezuelae* cultures was performed similarly to a method described in our previous report [14] with modifications—see below.

Briefly, the spores of Sven_ΔadpA/adpA-FLAG and Sven_ΔadpA strains (final OD = 0.001) were inoculated to GYM medium (malt extract 10 g/L, yeast extract 4 g/L, glucose 4 g/L, CaCl_2_ 1.46 g/L; pH 7.3) (50 mL) and grown in two independent replicates for 3 days, at 30 °C with shaking (140 or 220 rpm) using 250-mL baffled flasks. After that time, the cultures were spun down, and the obtained supernatants were additionally cleared through a filter paper. The supernatants were extracted twice with ethyl acetate added at a 1:1 ratio using a separating funnel. Upon extraction, the organic fractions obtained from the same sample were combined and dried using a rotary evaporator. The control samples were prepared by processing a sterile GYM medium in the way described above. The resultant crude extracts were dissolved in high-performance liquid chromatography (HPLC)-grade DMSO and stored in glass vials at −20 °C until further HPLC or liquid chromatography-mass spectrometry (LC-MS) analysis.

### 3.6. High-Performance Liquid Chromatography

HPLC was performed with a Shimadzu LC-2050C system (Kyoto, Japan). Before the analysis, the metabolite extracts were centrifuged to remove any solid particles. Samples (10 μL) were injected onto a column (ARION Plus C18, 3 μm, 150 mm × 3.0 mm) equilibrated with 5% B and separated with eluent A (0.1% formic acid in water) and eluent B (0.1% formic in acid acetonitrile) by gradient elution (5–100% B over 45 min) followed by isocratic elution at 100% B over 5 min at a flow rate of 400 mL/min. Detection was carried out at the wavelength range of 190–800 nm. The concentration of Cml in each extract was estimated by HPLC using the default Shimadzu LabSolutions software (version 5.124) and a standard calibration curve, with Cml (Carl Roth, Karlsruhe, Germany; 3886.3) (linearity range used for quantification 0.5–3 μg) eluting at approximately 21.2 min. Each set of analyzed samples included a blank solvent and a Cml standard run for confirmation.

### 3.7. Liquid Chromatography-Mass Spectrometry

LC-MS analysis was performed on the samples prepared for HPLC analysis using an M-Class Acquity UPLC system coupled to a Synapt XS high-resolution mass spectrometer (HRMS) equipped with an electrospray ionization (ESI) source (Waters, Milford, MA, USA). Mobile phase A consisted of H_2_O + 0.1% formic acid (FA), while mobile phase B consisted of acetonitrile + 0.1% FA (all solvents and additives were LC-MS grade and acquired through WITKO, Łódź, Poland). Before system placement bacterial extracts were diluted 50 times with H_2_O + 0.1% formic acid and 5 µL of each sample was injected, desalted on-system, and a 30 min 10–90% B linear gradient was applied for sample separation on an Acquity BEH C18 130 Å, 1.7 μm, 1 mm × 100 mm column (Waters), which was kept at 60 °C. MS data were collected in data-independent acquisition (DIA) mode (MS^E^) at 0.5 s/scan through a 50–1200 m/z range in time-of-flight (TOF) resolution mode. A collision-induced dissociation (CID) energy ramp of 20–40 V was applied for the fragmentation function. Samples were run in analogous methods using positive and negative polarity. A Leucine–Enkephalin solution (Waters, Milford, MA, USA) was acquired in the reference function, and the correction was applied in acquisition.

### 3.8. LC-MS Data Analysis

For all compounds except chloramphenicol, LC-MS data were manually analyzed in MassLynx (version 4.1) (Waters, Milford, MA, USA). MS1 ion peak matching tolerance assumed was below 10 ppm, while for MS2, a broader tolerance of 25 ppm was used. Isotopic profile consistency was checked for each compound. For MS2 data, fragmentation information was obtained from various freely available sources (as indicated in Table S7). XIC extraction windows were set to 15 ppm. For chloramphenicol ESI-analysis, precursor isolation windows were generated using MS^E^ Data Viewer (version 2.0) (Waters, Milford, MA, USA) and the top 10 ions of the 321.007 m/z, 16.3 min. RT ion window was passed to the massbank.eu databank search, which returned a high-confidence match.

## 4. Conclusions

Our findings expand the current model of AdpA-mediated regulation in Actinobacteria by defining, for the first time, the direct AdpA regulon in *S. venezuelae* and uncovering its broad regulatory influence on primary metabolism, secondary metabolism, and development.

This study establishes AdpA as a global regulator in *S. venezuelae*, orchestrating gene expression across both developmental and metabolic pathways. Through integrated RNA-seq and ChIP-seq analyses, AdpA was shown to directly bind to nearly 200 promoter regions and exert indirect regulatory influence over thousands of genes, including all annotated SM-BGCs. A core AdpA regulon comprising 49 to 91 genes—depending on the developmental stage—was identified. A notable example is AdpA’s activation of chloramphenicol biosynthesis, achieved by targeting multiple regulatory sites within the corresponding gene cluster. Deletion of *adpA* leads to a significant reduction in chloramphenicol production, confirming its functional importance. Furthermore, our data suggest that AdpA may compete with Lsr2 at overlapping binding sites, leading to derepression of the cluster. We also demonstrate that AdpA_Sv_ directly influences the biosynthesis of desferrioxamine, coproporphyrin, and melanin, resulting in several-fold changes in their production.

The regulatory impact of AdpA is dynamic and growth-stage dependent, with broader effects observed during aerial hyphae formation. This shift likely reflects a combination of direct DNA binding and indirect modulation via other transcription factors, as inferred from AdpA-dependent changes in their expression, and possibly chromosomal structural changes, as suggested by previous studies.

Motif analysis revealed a conserved AdpA-binding core with a previously unrecognized 3′ extension, suggesting more refined promoter specificity than previously appreciated.

Given its broad regulatory scope and ability to activate silent or weakly expressed SM-BGCs, AdpA emerges as a powerful tool for unlocking cryptic metabolic pathways. This positions AdpA not only as a key regulator but also as a promising lever in combinatorial biosynthetic strategies aimed at expanding the natural product repertoire, particularly for antimicrobial discovery.

## Data Availability

The ChIP-seq and RNA-seq datasets obtained in this study are available in the Array Express database (EMBL-EBI) under accession numbers E-MTAB-15314 and E-MTAB-15315, respectively.

## Supplementary Materials

The following supporting information can be downloaded at: https://www.mdpi.com/article/10.3390/antibiotics14090878/s1, Figure S1: Distribution of gene expression changes in KEGG pathways regulated by AdpA; Figure S2: Genes most affected by AdpA deletion; Figure S3: Coverage levels of *adpA* transcript in RNA-seq; Figure S4: Peak size distribution plots of MACS peaks for ChIP-seq results; Figure S5: Distribution of MACS peaks across the *S. venezuelae* chromosome in individual ChIP-seq samples; Figure S6: AdpA binding within the chloramphenicol biosynthetic gene cluster; Figure S7: Heatmap showing relative abundance of compounds identified in culture extracts using LC-MS; Figure S8: Detection of chloramphenicol in culture extracts; Table S1: Strains and primers used in the study; Table S2: Differentially expressed genes in *S. venezuelae*; (A) Differentially expressed genes in Sven_ΔadpA compared to the Sven_ΔadpA/adpA-FLAG strain at 12 hrs; (B) Differentially expressed genes in Sven_ΔadpA compared to the Sven_ΔadpA/adpA-FLAG strain at 20 hrs; (C) Common differentially expressed genes at 12 and 20 hr time points; (D) Genes most affected by AdpA deletion at 12 h; (E) Genes most affected by AdpA deletion at 20 h; (F) Differential expression of *Streptomyces* developmental genes; (G) Differentially expressed genes in Sven_ΔadpA compared to the Sven_ΔadpA/adpA-FLAG strain at 12 hrs under oxygen-depleted conditions; Table S3. ChIP-seq analysis. (A) MACS peak calling of ChIP-seq data forAdpA binding at the 12-h time point. (B) MACS peak calling of ChIP-seq data forAdpA binding at the 20-h time point. (C) Genomic locations of AdpA-binding motifs in *S. venezuelae* identified at the 12-h time point. (D) Genomic locations of AdpA-binding motifs in *S. venezuelae* identified at the 20-h time point. (E) Gene promoters with AdpA-binding motifs common to both the 12- and 20-h time points. (F) Genome-wide in silico identification of relaxed AdpA-binding motifs in *S. venezuelae*. (G) Putative direct AdpA regulon based on relaxed binding motif presence and expression data; Table S4: Differential expression of global regulators; (A) Differential expression of global regulators and secondary metabolism-associated transcriptional regulators; (B) Differential expression of Rex and WhiAB regulons; Table S5: Combined RNA-seq and ChiP-seq data for *S. venezuelae* biosynthetic gene clusters; Table S6: LC-MS detection of selected secondary metabolites in Sven_ΔadpA and Sven_ΔadpA/adpA-FLAG strains; Table S7: Functions of genes involved in chloramphenicol (Cml) biosynthesis and Cml-BGC gene expression (RPKM); Supplementary text.
