# Supplementary material for: AdpA, a Global Regulator of Hundreds of Genes, Including Those for Secondary Metabolism, in Streptomyces venezuelae
Source: Antibiotics (Basel). 2025 Aug 30;14(9):878. doi: 10.3390/antibiotics14090878 (PMC12466573; doi:10.3390/antibiotics14090878)
Supplement: Supplementary file 1 [file antibiotics-14-00878-s001.zip › SI_text_FIN.pdf]

AdpA, A Global Regulator of Hundreds of Genes, Including Those for Secondary Metabolism, in *Streptomyces venezuelae*

Authors:
Marcin Wolański*, Małgorzata Płachetka, Volha Naumouskaya, Agnieszka Strzałka, Michał Tracz, Diana Valietova and Jolanta Zakrzewska-Czerwińska

* corresponding author, marcin.wolanski@uwr.edu.pl

Faculty of Biotechnology, University of Wrocław, 50-383 Wrocław, Poland.

<u>The list of supplementary materials:</u>

1. Supplementary figures

Fig. S1. Distribution of gene expression changes in KEGG pathways regulated by AdpA.
Fig. S2. Genes most affected by AdpA deletion.
Fig. S3. Coverage levels of *adpA* transcript in RNA-seq.
Fig. S4. Peak size distribution plots of MACS peaks for ChIP-seq results.
Fig. S5. Distribution of MACS peaks across the *S. venezuelae* chromosome in individual ChIP-seq samples.
Fig. S6. AdpA binding within the chloramphenicol biosynthetic gene cluster.
Fig. S7. Heatmap showing relative abundance of compounds identified in culture extracts using LC-MS.
Fig S8. Detection of chloramphenicol in culture extracts.

2. Supplementary tables

Table S1. Strains and primers used in the study. (<u>see below in this file</u>)
Table S2. Differentially expressed genes in *S. venezuelae.* (<u>see in SI Excel file</u>)

- **A.** Differentially expressed genes in Sven_ΔadpA compared to the Sven_ΔadpA/adpA-FLAG strain at 12 hrs.
- **B.** Differentially expressed genes in Sven_ΔadpA compared to the Sven_ΔadpA/adpA-FLAG strain at 20 hrs.
- **C.** Common differentially expressed genes at 12 and 20 hr time points.
- **D.** Genes most affected by AdpA deletion at 12 hours.
- **E.** Genes most affected by AdpA deletion at 20 hours.
- **F.** Differential expression of *Streptomyces* developmental genes.
- **G.** Differentially expressed genes in Sven_ΔadpA compared to the Sven_ΔadpA/adpA-FLAG strain at 12 hrs under oxygen-depleted conditions.

Table S3. ChIP-seq analysis. (<u>see in SI Excel file</u>)

- **A.** MACS peak calling of ChIP-seq data forAdpA binding at the 12-hour time point.
- **B.** MACS peak calling of ChIP-seq data forAdpA binding at the 20-hour time point.
- **C.** Genomic locations of AdpA-binding motifs in *S. venezuelae* identified at the 12-hour time point.
- **D.** Genomic locations of AdpA-binding motifs in *S. venezuelae* identified at the 20-hour time point.
- **E.** Gene promoters with AdpA-binding motifs common to both the 12- and 20-hour time points.
- **F.** Genome-wide in silico identification of AdpA-binding motifs in *S. venezuelae*.
- **G.** Putative direct AdpA regulon based on binding motif presence and expression data.

Table S4. Differential expression of global regulators. (<u>see in SI Excel file</u>)

- **A.** Differential expression of global regulators and secondary metabolism-associated transcriptional regulators.
- **B.** Differential expression of Rex and WhiAB regulons.

Table S5. Combined RNA-seq and ChiP-seq data for *S. venezuelae* biosynthetic gene clusters. (see in SI Excel file)

Table S6. LC-MS detection of selected secondary metabolites in Sven_ΔadpA and Sven_ΔadpA/adpA-FLAG strains. (see in SI Excel file)

Table S7. Functions of genes involved in chloramphenicol (Cml) biosynthesis and Cml-BGC gene expression (RPKM). (see in SI Excel file)

3. Supplementary Materials and Methods
Cultures in oxygen-depleted conditions
Protein-DNA interactions
Internet links to sequence resources and bioinformatic tools

4. Supplementary References
See the bottom of this file.

Supplementary figures

Fig. S1. Distribution of gene expression changes in KEGG pathways regulated by AdpA.

The KEGG ridge plot shows expression dynamics of AdpA-associated genes with enriched metabolic pathways (ridges) that are either activated (ridge shifted to the right) or suppressed (ridge shifted to the left) in the Sven_ΔadpA compared to the Sven_ΔadpA/adpA-FLAG strain at 12 **(A)** and 20-hour **(B)** time points. The genes were grouped according to the KEGG database. The enrichment distribution refers to log_2FC change. The pathways with higher peaks indicate a larger density of genes (similar enrichment scores) with significant changes (based on the adjusted p-value). Gene grouping analysis was performed in R using ClusterProfiler package.

Fig. S2. Genes most affected by AdpA deletion.

Volcano plots showing the relationship between gene expression changes (log_2 fold change) and statistical significance ($-log_{10}$ *P*-value) based on RNA-seq data comparing Sven_ΔadpA and Sven_ΔadpA/adpA-FLAG strains at 12-hour **(A)** and 20-hour **(B)** time points. Genes most strongly up- or downregulated (based on log_2 fold change) are labeled (see Table S2D-E). Plots were generated using a custom R script.

Fig. S3. Coverage levels of *adpA* transcript in RNA-seq.

The plots show *adpA* (vnz_12630) transcript coverage from combined RNA-seq data of three biological replicates for the Sven_Δ*adpA* and Sven_Δ*adpA*/adpA-FLAG strains at 12- and 20-hour time points.

Fig. S4. Peak size distribution plots of MACS peaks for ChIP-seq results.

The distribution of MACS-called peaks at the 12 and 20-hour time points (left and right panels, respectively). The X-axis represents peak lengths, the Y-axis indicates the number of peaks, and the red dotted vertical line indicates an average peak length for the corresponding set.

Fig. S5. Distribution of MACS peaks across the *S. venezuelae* chromosome in individual ChIP-seq samples.

The plots display the distribution of MACS peaks for all samples used in the ChIP-seq experiment. The samples in the panel include:

- FLAG_12_1 and FLAG_12_2: Biological replicates of immunoprecipitated 12-hour samples (crosslinked, precipitated, and purified on affinity resin) from the AdpA-FLAG-tagged strain (*Sven_ΔadpA/adpA-FLAG*).
- FLAG_12_IN_1 and FLAG_12_IN_2: Biological replicates of 12-hour "input" samples (crosslinked and precipitated) from the AdpA-FLAG-tagged strain.
- FLAG_20_1 and FLAG_20_2: Biological replicates of immunoprecipitated 20-hour samples for the AdpA-FLAG-tagged strain.
- FLAG_20_IN_1 and FLAG_20_IN_2: Biological replicates of 20-hour "input" samples for the AdpA-FLAG-tagged strain.

WT Samples: "Input" samples collected at the 12-hour and 20-hour time points from the wild-type strain (Sven_WT) cultures, serving as control non-FLAG samples.

Fig. S6. AdpA binding within the chloramphenicol biosynthetic gene cluster.

(**A**) Electrophoretic mobility shift assay (EMSA) using SacI-digested 4O20 fosmid DNA (StrepDB) incubated with increasing concentrations of AdpASv_His protein (100 and 500 nM). DNA bands exhibiting altered migration patterns compared to the protein-free control are indicated by arrows: orange for weak shifts and red for strong shifts. M – DNA molecular weight marker (GeneRuler 1 kb DNA Ladder, Thermo Fisher Scientific). The right panel maps the positions of the shifted DNA fragments (dotted lines) on the 4O20 cosmid. **(B)** EMSA with PCR-amplified DNA fragments corresponding to region 3 identified in panel A. The full-length fragment (fragment 1, red dotted line), a subfragment (fragment 2, blue dotted line), and a negative control (NC) were incubated with increasing concentrations of AdpASv_His (10, 50, and 250 nM; indicated by black triangles). Yellow arrows mark free DNA, showing progressive depletion corresponding to specific protein binding.

Fig. S7. Heatmap showing relative abundance of compounds identified in culture extracts using LC-MS.

The heatmap represents Z-score normalized peak area intensities of LC-MS features corresponding to detected compounds. Each row corresponds to a distinct compound, and each column represents strains and conditions. Normalization was performed to highlight relative differences in compound abundance across samples. Compounds were putatively identified based on accurate mass. Color scale represents Z-scores of peak area intensities, with red indicating higher and blue indicating lower relative abundance.

Fig. S8. Detection of chloramphenicol in culture extracts.

(A) Chloramphenicol (MH−=321.0045) identity in the Sven_ΔadpA/adpA-FLAG strain sample was confirmed by MS1 and MS2 to be the ~16.3 min. RT peak. Chloramphenicol fragmentation data match was obtained by searching the top 10 ions of the MS2 isolation window for the 321.007 precursor ion in the massbank.eu database with a 0.9 score threshold. Best matches were obtained for chloramphenicol accessions MSBNK-BAFG-CSL23111012409 and MSBNK-Eawag-EQ307053 (scores of 0.92 and 0.91, respectively). Most intense fragment ions were annotated. **(B)** The relative amount of chloramphenicol, as judged by the extracted ion chromatogram (XIC), was ~30 times greater in the Sven_ΔadpA/adpA-FLAG strain, than in the Sven_ΔadpA strain.

Supplementary tables

Table S1. Strains and primers used in the study.

**Strain**	**Genotype (description)**	**Reference/source**
	Streptomyces venezuelae	
Sven_WT	Wild-type *S. venezuelae* NRRL B-65442 strain	[1]; John Innes Centre, Norwich UK
Sven_ΔadpA	NRRL B-65442 *adpA::aac(3)IV* (*adpA* deletion mutant)	[2]
Sven_ΔadpA/adpA-FLAG	Sven_ΔadpA::pMS83-adpA-3xFLAG (a*dpA* deletion mutant complemented with *adpA-FLAG*)	[2]
**Primer name**	**Sequence**	**Purpose**
E1_Sac1368_fw_1	AGCTCCCATGAATTGTCCCAGG	Amplification of DNA fragment 1 for EMSA (Figure S6B)
E1_Sac1368_fw_2	ATGTTCACGATCGCCCCG	Amplification of DNA fragment 2 for EMSA (Figure S6B)
E1_Sac1368_rv_1	AGCTCGGCAAGTTCGGCA	Amplification of DNA fragments 1 and 2 for EMSA (Fig. S6B)
pTZCy5	(Cy5)-TCGGTACCTCGCGAATGCATC	Amplification of DNA fragment NC for EMSA (Figure S6B) (pdN fragment from the previous study (Płachetka et al)
hp0500_EcoRV_Fd	CTGATATCATGAAAATCAGTGTTAGTAAAA AC	Amplification of DNA fragment NC for EMSA (Figure S6B) (pdN fragment from the previous study [2]

Supplementary Materials and Methods

Cultures in oxygen-depleted conditions

The cultures were grown under the conditions described in the Materials and Methods section of the manuscript, under 'Chromatin immunoprecipitation-sequencing and bioinformatics analysis'. Oxygen-depleted conditions were induced at 11 hours by blowing nitrogen gas into the upper part of the culture flask for 10–15 seconds using a hose connected to a 0.22 µm filter. The flasks were then re-capped, sealed with Parafilm, and additionally wrapped with a rubber laboratory glove to prevent gas exchange. The cultures were maintained under these conditions, and samples were collected at the 12-hour time point.

Protein purification

Recombinant AdpASv_His protein was purified following the protocol previously described [2], with no significant modifications.

Protein-DNA interactions

EMSA assays with restriction-digested cosmid

Protein–DNA interaction studies between recombinant AdpASv_His and restriction-digested cosmid 4O20 comprising the chloramphenicol gene cluster (genome range: 1014350-1057188) were performed as previously described [3], with minor modifications. Cosmid 4O20 DNA (StrepDB) was isolated using the Plasmid Maxi AX kit (A&A Biotechnology). The purified DNA was digested overnight at 37°C with a restriction enzyme (New England Biolabs), followed by thermal inactivation of the enzyme according to the manufacturer's instructions.

Master mixes were prepared containing 750 ng of digested DNA, HBS200 buffer, BSA (5 µg/µL), and nuclease-free water. Aliquots of 18 µL were transferred into individual tubes and combined with 2 µL of recombinant AdpASv_His protein diluted in A100 buffer (50 mM NaH_2PO_4, pH 7.4; 300 mM NaCl; 100 mM imidazole; 10% glycerol) to final concentrations of 100 nM and 500 nM. A control reaction containing only A100 buffer (no protein) was included as a negative control. Binding reactions were incubated at room temperature for 30 minutes.

Following incubation, 20 µL of each sample was loaded onto a 1% agarose gel prepared in 0.5× TBE buffer. Electrophoresis was conducted overnight at 20 V at room temperature using the same buffer. After electrophoresis, the gel was stained with ethidium bromide for 30 minutes and visualized using the ChemiDoc MP imaging system (Bio-Rad). DNA migration patterns were analyzed using the GeneRuler 1 kb DNA Ladder (Thermo Scientific) as a molecular weight reference.

EMSA assays with PCR-amplified DNA fragments

Protein–DNA binding assays were performed with minor modifications to the protocol described in the previous study [2].

Briefly, DNA fragments were first amplified by PCR. The resulting reaction mixtures were treated with thermosensitive alkaline phosphatase and Exonuclease I (Thermo Fisher Scientific) to remove residual primers and single-stranded DNA. Following enzymatic digestion, DNA was purified using the CleanUp Concentrator kit (A&A Biotechnology).

For EMSA, recombinant AdpASv_His protein was diluted to final concentrations of 10 nM, 50 nM, and 250 nM in A100 buffer (50 mM NaH_2PO_4, pH 7.4; 300 mM NaCl; 100 mM imidazole; 10% glycerol). Master mixes were prepared containing 20 ng of DNA, 5 µg/µL BSA (in 5% glycerol), HBS200 buffer (10 mM HEPES, pH 7.4; 10 mM MgOAc; 200 mM NaCl; 3.4 mM EDTA; 0.05% Tween 20; 5% glycerol), and nuclease-free water. Each 18 µL master mix was combined with 2 µl of the appropriate protein dilution or A100 buffer (control) and incubated at room temperature for 30 minutes.

Samples were resolved on a 4% polyacrylamide gel pre-run in 0.25× TBE buffer at 100 V for 30 minutes at 4–8°C. Electrophoresis was carried out at 100 V for 4 hours under the same temperature conditions. DNA was visualized post-run by ethidium bromide staining using the ChemiDoc MP imaging system (Bio-Rad).

Internet links to sequence resources and reference numbers to omics data sets

1. **Deposited RNA-seq data**
   The RNA-Seq data sets obtained for normal and oxygen-limited conditions produced in this study are available in the Array Express database (EMBL-EBI) (https://www.ebi.ac.uk/biostudies/arrayexpress) under accession numbers **E-MTAB-15315**.

2. **Deposited ChIP-seq data**
   The ChIP-seq data sets obtained in this study are available in the Array Express database (EMBL-EBI) (https://www.ebi.ac.uk/biostudies/arrayexpress) under accession numbers **E-MTAB-15314**.

3. ***Streptomyces venezuelae* NRRL B-65442 chromosome sequence**
   The genome sequence of *S. venezuelae* used in this study (NCBI Reference Sequence NZ_CP018074.1) and gene annotation files are directly accessible under the following link: https://ftp.ncbi.nlm.nih.gov/genomes/all/GCF/001/886/595/GCF_001886595.1_ASM188659v1/

   The genome assembly file "GCA_001886595.1_ASM188659v1_genomic.gbff" available under the above link was used to identify biosynthetic gene clusters using antiSMASH.

4. **The Streptomyces Annotation Server (StrepDB)**
   https://strepdb.streptomyces.org.uk/

5. **Bioinformatics tools**

Name	Internet address	Description (references)
MEME-ChIP	https://meme-suite.org/meme/tools/meme-chip	Motif discovery in ChIP-seq data [4,5]
FIMO	https://meme-suite.org/meme/tools/fimo	Sequence scanning for motif occurrence [6]
R	https://www.r-project.org/	RNA-seq and ChIP-seq data analysis
LogoMotif	https://logomotif.bioinformatics.nl/	Database of transcription regulators binding sites [7]

Supplementary references

1. Gomez-Escribano, J.P.; Holmes, N.A.; Schlimpert, S.; Bibb, M.J.; Chandra, G.; Wilkinson, B.; Buttner, M.J.; Bibb, M.J. Streptomyces Venezuelae NRRL B-65442: Genome Sequence of a Model Strain Used to Study Morphological Differentiation in Filamentous Actinobacteria. *J Ind Microbiol Biotechnol* **2021**, *0*, 35, doi:10.1093/jimb/kuab035.

2. Płachetka, M.; Krawiec, M.; Zakrzewska-Czerwińska, J.; Wolański, M. AdpA Positively Regulates Morphological Differentiation and Chloramphenicol Biosynthesis in *Streptomyces Venezuelae*. *Microbiol Spectr* **2021**, *9*, e01981-21, doi:10.1128/spectrum.01981-21.

3. Wolański, M.; Łebkowski, T.; Kois-Ostrowska, A.; Zettler, J.; Apel, A.K.; Jakimowicz, D.; Zakrzewska-Czerwińska, J. Two Transcription Factors, CabA and CabR, Are Independently Involved in Multilevel Regulation of the Biosynthetic Gene Cluster Encoding the Novel Aminocoumarin, Cacibiocin. *Appl Microbiol Biotechnol* **2016**, *100*, 3147–3164, doi:10.1007/s00253-015-7196-7.

4. Machanick, P.; Bailey, T.L. MEME-ChIP: Motif Analysis of Large DNA Datasets. *Bioinformatics* **2011**, *27*, 1696–1697, doi:10.1093/BIOINFORMATICS/BTR189.

5. Bailey, T.L.; Boden, M.; Buske, F.A.; Frith, M.; Grant, C.E.; Clementi, L.; Ren, J.; Li, W.W.; Noble, W.S. MEME Suite: Tools for Motif Discovery and Searching. *Nucleic Acids Res* **2009**, *37*, W202–W208, doi:10.1093/nar/gkp335.

6. Grant, C.E.; Bailey, T.L.; Noble, W.S. FIMO: Scanning for Occurrences of a given Motif. *Bioinformatics* **2011**, *27*, 1017–1018, doi:10.1093/BIOINFORMATICS/BTR064.

7. Augustijn, H.E.; Karapliafis, D.; Joosten, K.M.M.; Rigali, S.; van Wezel, G.P.; Medema, M.H. LogoMotif: A Comprehensive Database of Transcription Factor Binding Site Profiles in Actinobacteria. *J Mol Biol* **2024**, *436*, 168558, doi:10.1016/J.JMB.2024.168558.
